# Paired proteomic analysis reveals protein alterations in sun-exposed skin of professional drivers

**DOI:** 10.1038/s41598-024-82308-8

**Published:** 2025-03-31

**Authors:** Amanda C. Camillo-Andrade, Lucas A. Sales, Juliana S. G. Fischer, Rosario Duran, Marlon D. M. Santos, Paulo C. Carvalho

**Affiliations:** 1 Laboratory for Structural and Computational Proteomics, Carlos Chagas Institute, Fiocruz, Rua Prof. Algacyr Munhoz Mader 3775, Curitiba, Paraná Brazil; 2 Analytical Biochemistry and Proteomics Unit, Instituto de Investigaciones Biológicas Clemente Estable, Institut Pasteur de Montevideo, Montevideo, Uruguay; 3 Positivo University, Paraná, Brazil

**Keywords:** Photoaging, Skin, Proteomics, Paired, Solar radiation, Biochemistry, Proteins, Proteome

## Abstract

Professional drivers represent an ideal cohort for investigating the effects of solar radiation on skin due to their unique, asymmetric exposure to sun, a consequence of vehicle window orientations. Consequently, one side of the face is naturally subjected to more solar radiation, resulting in uneven sunlight exposure. This scenario supports a paired experimental design for precise within-individual comparisons, crucial for assessing sun exposure’s impact on skin health, including signs of aging. Leveraging this approach, our study reveals sun-induced overexpression of proteins linked to photoaging through paired proteomic analysis, providing novel insights into the skin’s adaptive responses to chronic solar exposure. Initially, our research focused on a dataset from ten male professional drivers, identifying a set upregulated proteins in sun-exposed skin compared to the less exposed side of the face. To validate these findings, we extended our investigation to a new cohort of seven female bus drivers. Our motivation in switching genders and utilizing different mass spectrometry equipment and sample preparation techniques was for assessing the robustness of our initial findings, encompassing not just sex differences but also methodological variations, and also for understanding the broader implications of our results for photodermatology. To enable this detailed analysis, we developed specialized software that allows precise paired proteomic analysis, significantly enhancing the robustness and clarity of our findings. Our results shortlisted keratins, S100A14, and F-box proteins—by remaining consistently overexpressed in sun-exposed skin—and hemoglobin subunit beta as downregulated across both cohorts. Our findings underscore the potential of proteomic techniques in advancing our understanding of the molecular dynamics of photoaging and highlight the value of selecting cohorts with specific exposure characteristics.

**Supplementary Information:**

The online version contains supplementary material available at 10.1038/s41598-024-82308-8.

## Introduction

The influence of the exposome on the skin, encompassing both internal and external factors, significantly influences its health and aging processes^1^. As the body’s largest organ and primary interface with the environment, the skin is continually exposed to various elements^2^, with solar radiation playing a critical role in its health and the acceleration of aging, known as photoaging^1^. This encompasses exposure to ultraviolet (UV) rays, visible light (VL), and infrared radiation (IRA), each contributing distinctively to skin conditions and aging signs like roughness, age spots, and pigmentation changes^3^. The damage induced by these radiation types, through the generation of reactive oxygen species, affects DNA, proteins, and lipids, altering skin structure and composition^1,4^. Understanding the complex impact of solar radiation on the skin within the context of the exposome is essential for maintaining skin health and mitigating the effects of aging.

Proteomics stands as a powerful tool in the identification and quantification of a vast array of proteins within complex biological matrices, such as body fluids, cell lysates, and tissue samples, including skin^5^. The application of proteomics in human studies establishes significant challenges, largely attributed to the huge variability inherent to the human population. The intricate complexity and diversity of the human proteome, coupled with the difficulty of acquiring consistent and representative samples, stand substantial obstacles^6^. These challenges are further amplified by the variability between individuals and the dynamic fluctuations in protein abundance triggered by an array of internal and external factors.

The strategic implementation of paired study designs in omics has proven essential in overcoming these hurdles, enabling more definitive and meaningful scientific discoveries. Such designs allow for the direct comparison of samples from the same individual under differing conditions, thereby mitigating the confounding effects of inter-individual variability and ensuring a more nuanced understanding of proteomic alterations^7^. Brunoro and colleagues employed proteomics, coupled with a paired experimental and statistical approach, to identify breast cancer biomarkers by analyzing nipple aspirate fluid from both cancerous and non-cancerous breasts of the same women^8^. This method, which compared affected and unaffected tissues within individuals, was pivotal in successfully isolating a panel of biomarkers. This effectively overcame the limitations encountered in previous attempts by the group, which had struggled to pinpoint such markers without the benefit of the paired approach.

Addressing the unique demands of paired proteomic approaches for increased sensitivity, as part of this manuscript, we also provide a software that can perform pairwise proteomic analysis and is directly compatible with PatternLab for proteomics^9^ identification results. This innovation is specifically tailored to enhance the accuracy of detecting proteins with differential abundance across varied conditions, such as comparing sun-exposed versus less exposed skin regions.

Here we utilized two datasets, the first comprising proteomic profiles from 10 male professional drivers and a subsequent one including seven female drivers. For each participant, skin samples were collected from both the left and right sides of the face. Through paired analysis of protein abundance variations in both male and female cohorts, independently, we shortlisted the proteins that were most impacted due to sun exposure.

## Results

### Proteomic identification and quantification overview

In the dataset from professional male drivers, we identified and quantified a total of 1,083 proteins and 11,972 peptides across all samples. For the professional female drivers, the dataset revealed a total of 1,502 proteins and 18,081 peptides in all samples. Details are available in the Supplementary Quantification Male and Female, respectively. The dataset concerning professional male drivers is elaborated upon in the data descriptor^10^, and the complete dataset can be accessed for download on the PRIDE^11^ database under the identifier PXD045887. Similarly, the validation dataset from professional female drivers is accessible for download on PRIDE, using the identifier PXD050746.

### Proteomic pairwise comparer availability

Proteomic Pairwise Comparer software is freely available for academic use athttp://patternlabforproteomics.org/ppa. A detailed tutorial and workflow is provided on the project website.

### Differentially abundant proteins

Tables 1 and 2 present a curated list of proteins identified as differentially abundant by our Proteomics Paired Comparer Sample Analyzer, with *p*-values determined using the widely accepted TFold module in PatternLab for proteomic data analysis. Standard practices often employ *p*-value cutoffs of 0.01 or 0.05; however, we intentionally opted for a more lenient cutoff of 0.10 in this study. This decision was motivated by our goal to encourage broader data exploration, aiming to uncover significant trends and patterns potentially overlooked under stricter criteria.

**Table 1:** Proteomic analysis of male Professional Drivers

Locus	Fold change (Log_2_)	Paired	T-Fold	Description
		*p*-value	*p*-value	
P35527	1.73	< 0.01	0.02	Keratin, type I cytoskeletal 9
Q5T749	1.77	< 0.01	0.04	Keratinocyte proline-rich protein
P13647	1.27	0.02	0.04	Keratin, type II cytoskeletal 5
P02538	1.06	0.03	0.12	Keratin, type II cytoskeletal 6 A
P29373	0.92	0.03	0.09	Cellular retinoic acid-binding protein 2
Q9HCY8	1.24	0.03	0.07	Protein S100-A14
P02533	1.01	0.03	0.07	Keratin, type I cytoskeletal 14
O76011	1.82	0.04	0.05	Keratin, type I cuticular Ha4
Q6ZVX7	0.66	0.04	0.23	F-box only protein 50
P49327	1.24	0.04	0.16	Fatty acid synthase
Q04695	0.87	0.04	0.11	Keratin, type I cytoskeletal 17
Q7Z794	1.01	0.05	0.27	Keratin, type II cytoskeletal 1b
P08779	0.93	0.05	0.08	Keratin, type I cytoskeletal 16
P68871	-2.31	0.07	0.43	Hemoglobin subunit beta
P31947	0.99	0.07	0.16	14-3-3 protein sigma
P15586	1.28	0.09	0.14	N-acetylglucosamine-6-sulfatase
Q9GZZ8	1.74	0.09	0.07	Extracellular glycoprotein lacritin
P19957	1.07	0.10	0.11	Elafin

This table lists proteins identified as differentially abundant between sun-exposed and non-exposed sides of the face. Fold Change is presented in Log2 format, with positive values indicating higher abundance on the sun-exposed side. The table includes protein locus, fold change, paired p-value (within subjects), T-Fold p-value (across subjects), and a brief description of each protein. The paired p-value is derived from our novel software, proteomic pairwise comparer, introduced in this manuscript, taking advantage of the within-subject variability, while the T-Fold p-value, is provided by PatternLab for Proteomics, a widely adopted proteomic data analysis software.

**Table 2:** Proteomic Analysis of Female Professional Drivers

Locus	Fold Change (Log_2_)	Paired	T-Fold	Description
		*p*-value	*p*-value	
Q9BYR8	-2.22	0.01	0.42	Keratin-associated protein 3 − 1
Q9UBM7	0.77	0.03	0.26	7-dehydrocholesterol reductase
P35527	1.10	0.03	0.36	Keratin, type I cytoskeletal 9
O75608	0.42	0.03	0.30	Acyl-protein thioesterase 1
P05387	0.72	0.04	0.29	Large ribosomal subunit protein P2
O76009	-1.42	0.04	0.07	Keratin, type I cuticular Ha3-I
P54652	-0.58	0.05	0.41	Heat shock-related 70 kDa protein 2
Q9NQR4	-0.57	0.05	0.25	Omega-amidase NIT2
O43790	-1.51	0.05	0.42	Keratin, type II cuticular Hb6
Q7Z794	0.71	0.05	0.26	Keratin, type II cytoskeletal 1b
P08574	0.39	0.07	0.21	Cytochrome c1, heme protein, mitochondrial
P04066	0.55	0.08	0.36	Tissue alpha-L-fucosidase
Q8N1N4	0.66	0.08	0.02	Keratin, type II cytoskeletal 78
Q8IUC1	-1.51	0.08	0.30	Keratin-associated protein 11 − 1
P68871	-1.01	0.08	0.45	Hemoglobin subunit beta
P50990	0.98	0.08	0.12	T-complex protein 1 subunit theta
P02649	-1.00	0.08	0.01	Apolipoprotein E
P04264	0.66	0.09	0.06	Keratin, type II cytoskeletal 1
Q6ZVX7	0.21	0.09	0.10	F-box only protein 50
P13667	-0.46	0.10	0.23	Protein disulfide-isomerase A4
Q15631	0.88	0.10	0.16	Translin
Q8NEX9	0.37	0.10	0.21	Short-chain dehydrogenase/reductase family 9 C member 7
P22695	0.55	0.10	0.44	Cytochrome b-c1 complex subunit 2, mitochondrial
Q7Z2K6	-0.78	0.10	0.27	Endoplasmic reticulum metallopeptidase 1
P17050	-0.28	0.10	0.40	Alpha-N-acetylgalactosaminidase
O00487	0.14	0.107	0.22	26 S proteasome non-ATPase regulatory subunit 14
Q9HCY8	0.63	0.107	0.25	Protein S100-A14
P13645	0.64	0.107	0.21	Keratin, type I cytoskeletal 10
P01024	0.58	0.108	0.10	Complement C3

Structured similarly to table 1, this table focuses on the female cohort, detailing proteins with differential abundance due to sun exposure. It encompasses protein locus, fold change in Log2, paired and T-Fold p-values, and protein descriptions

The proteins identified as differentially abundant in both the male and female cohorts, and shared between Tables 1 and 2, include Keratin, type I cytoskeletal 9 (P35527), Keratin, type II cytoskeletal 1b (Q7Z794), Hemoglobin subunit beta (P68871), F-box only protein 50 (Q6ZVX7), and Protein S100-A14 (Q9HCY8). Of these, only hemoglobin was found to have decreased abundance in areas exposed to the sun.

Supplementary File 1 provides a spreadsheet shortlisting all the identified proteins in both datasets.

## Discussion

### Independent cohort validation strengthens evidence for proteins involved in photoaging

Our initial data analysis strategy adopted a *p*-value threshold of 0.1; we argue this to be a strategic decision as we aimed to broadening the scope of our investigation to include proteins with potential relevance to the skin’s response to solar exposure and photoaging. While photoaging-related changes such as solar elastosis typically occur within the dermis, the epidermis reflects cumulative proteomic alterations resulting from chronic sun exposure and is influenced by proteins synthesized in the dermis. Therefore, we postulate that analyzing the epidermis provides a non-invasive and painless method to capture key aspects of the skin’s adaptive response to photoaging. The subsequent validation of these proteins exhibiting similar *p*-values in an independent cohort significantly strengthens the argument for their biological significance. This dual observation of *p*-values below our 0.1 threshold across independent studies reduces the likelihood that our findings are mere artifacts of random chance. Our panel is further strengthened as we once again note that the datasets of these cohorts were generated from donors of different sex and the data was generated with different mass spectrometers; one using the Orbitrap Fusion Lumos and the other, an Exploris 240.

We turn to the statistical methodology known as Fisher’s Combined Probability Test, to further solidify our protein panel, once shortlisting the common proteins. This approach allows for the amalgamation of *p*-values from multiple independent tests assessing the same hypothesis, providing a mechanism to synthesize cumulative evidence. In other words, this test operates under the principle that the collective evidence from multiple independent studies can be synthesized to provide a unified measure of significance, thereby enhancing the overall interpretative value of the individual studies’ outcomes.

When applied to our context, where similar *p*-values were observed in two distinct cohorts, Fisher’s method mathematically combines these probabilities. The resulting combined *p*-value offers a more compelling statistical argument for the significance of the proteins’ roles in photoaging than any single study’s *p*-value could. Importantly, Fisher’s method is particularly suited to our situation because it assumes independence between the tests (conducted in separate cohorts). Within this framework, all shortlisted proteins achieve statistical significance (p-value < 0.05) when combining both p-values with the Fischer’s Combined Probability Test. For instance, even in a conservative scenario where a protein has exactly two p-values of 0.1, Fisher’s method would yield a combined *p*-value of approximately 0.057, which, although marginally above the commonly used threshold of 0.05, still suggests a trend worthy of further investigation.

### Increased abundance of S100-A14 in sun-exposed skin could be linked with cellular defense and stress response

The S100A14 (Q9HCY8) protein is a member of the S100 protein family, characterized by their calcium-binding capabilities and involvement in various cellular functions, including cell proliferation and responding to oxidative stress—two critical components for maintaining skin homeostasis^12^. As part of the skin’s complex signaling network, S100-A14 contributes to the mechanisms that accelerate skin healing and regeneration after cellular damage. S100A14 is involved in key pathways related to skin aging and repair, particularly through the regulation of matrix metalloproteinases (MMPs) that degrade extracellular matrix components^13^. By modulating MMP activity, S100A14 may influence extracellular matrix remodeling and contribute to the skin’s adaptive response to chronic sun exposure. The increase in S100-A14 abundance in areas of the skin exposed to chronic sun suggests its connected role in reinforcing the skin’s defenses against environmental stressors.

### F-box only protein 50 suggests modulation of protein stability and stress response

F-box only protein 50 (QCZVX7) is a component in the ubiquitination process that leads to protein degradation. This mechanism is essential for regulating various cellular processes, including cell cycle progression, response to stress, and maintenance of protein homeostasis^14^. In the context of skin, the ubiquitination system, facilitated by proteins like FBXO50, is essential for adapting to and mitigating damage from environmental stressors^15^ such as solar radiation. F-box proteins, such as FBXO50, play a crucial role in the ubiquitin-proteasome system, which is essential for protein degradation and turnover^16^. Through this pathway, F-box proteins may regulate the stability of proteins involved in cell cycle control and stress responses, thereby impacting skin aging and repair mechanisms in response to UV-induced damage.

The abundance of FBXO50 is increased on the side of the face more exposed to the sun, suggesting an adaptation of the skin to chronic solar exposure. This adaptation likely involves the modulation of the protein degradation pathway to manage damaged or misfolded proteins resulting from solar-induced stress. By promoting the selective degradation of damaged proteins, FBXO50 may help maintain cellular integrity and prevents the accumulation of cellular debris, thereby aiming to mitigate the effects of premature aging signs associated with sun exposure.

### Synergistic roles of keratin types I and II in skin resilience against solar radiation

Keratin, types I cytoskeletal 9 (P35527) and II cytoskeletal 1b (Q7Z794), are crucial for maintaining the skin’s structural integrity and resilience, especially against solar radiation^17,18^. These proteins support keratinocyte differentiation and strengthen the stratum corneum, the skin’s primary barrier^19,20^. Our findings show that sun exposure significantly increases P35527 and Q7Z794 levels, enhancing the stratum corneum’s density and its ability to protect against environmental damage. This response is essential for maintaining the epidermis’s health under UV stress^21^.

## Hemoglobin downregulation linked to sun exposure adaptation

Hemoglobin, traditionally recognized for its role in oxygen transport within erythrocytes, has recently been identified in non-erythroid sites, including skin keratinocytes, suggesting a broader functional repertoire beyond oxygen carriage^22^. While our results point toward a down-regulation in chronic-sun exposure, we must note that there are papers that report otherwise; nonetheless with data originating from mRNA reads in murine^22^. We hypothesize that the observed decrease in hemoglobin in sun-exposed skin is directly linked to its role in redox reactions, a response to specific environmental conditions like solar radiation^23,24^. Upon exposure to sunlight, hemoglobin has the potential to catalyze the formation of reactive oxygen species (ROS), which are molecules known for their damaging effects on cellular components, including DNA, proteins, and lipids^23^. This oxidative stress can lead to cellular aging, inflammation, and even carcinogenesis, particularly in skin chronic exposed to solar radiation. Given this mechanism, the downregulation of hemoglobin in areas exposed to the sun may represent a cellular adaptation aimed at minimizing the generation of harmful ROS. By reducing hemoglobin levels, skin cells could potentially lower the risk of solar radiation-induced oxidative damage, aligning with a protective strategy against the deleterious effects of continuous sun exposure.

## Materials and methods

This study’s methodology was approved by the Fiocruz Research Ethics Committee (approval number: CAAE 38352020.8.0000.5248) and involved the participation of Caucasian individuals with skin phototypes II to IV. The male participants, whose data we contrast in this work, had ages ranging from 37 to 54 years. The female participants included in this study had ages ranging from 34 to 55 years. The exclusion criteria include individuals with skin diseases, smokers, or individuals with diabetes. All methods were performed in accordance with relevant guidelines and regulations, with written informed consent obtained from all participants.

Skin samples were collected from both sides of the face using a microdermabrasion-like technique; thus, each participant provided two samples. Prior to sample collection, the skin was thoroughly cleaned with a cotton pad soaked in micellar water to remove surface contaminants and excess oils.

The sample preparation and mass spectrometry data acquisition were performed exactly as previously detailed by us^10^. Proteomic identification was performed using PatternLab for proteomics V software. Peptide identification was based on the SwissProt Homo sapiens sequence database and bioinformatic analysis, including protein quantitation by Extracted Ion Chromatogram (XIC) was performed exactly as previously described^9,25,26^.

### Comprehensive proteomic analysis across gender and equipment variances

This manuscript builds upon two distinct cohorts. The first, consists of 20 samples: 10 from the left and 10 from the right side of the facial skin of male bus drivers in Curitiba, Brazil^10^. The second, serving for validation purposes, comprising seven female drivers from the same city, contributing an additional 14 samples in total. The primary distinction in data generation between the cohorts lies in the mass spectrometry equipment used: the initial dataset was produced using an Exploris 240 Mass Spectrometer, whereas the subsequent dataset utilized an Orbitrap Fusion Lumos. Aside from this, the protocols were the same; therefore, mitigating experimental variability that could arise from differing sample preparation procedures.

### Enhancing proteomic analysis through sample pairing

To enable paired proteomic statistics, we generated a software named Proteomic Pairwise Comparer. Typical proteomic data analyses calculate fold changes and *p*-values for each protein across different conditions or treatments to identify those that are differentially expressed. However, these methods frequently overlook the natural variability seen in measurements taken from the same person, a factor that’s especially important in studies where people are compared against their own baseline. Our “Paired Sample Analyzer” software addresses this limitation by allowing the user to pair samples from the same individual under different conditions, thereby providing a more accurate and nuanced statistic of protein abundancy changes.

**Fig. 1:**
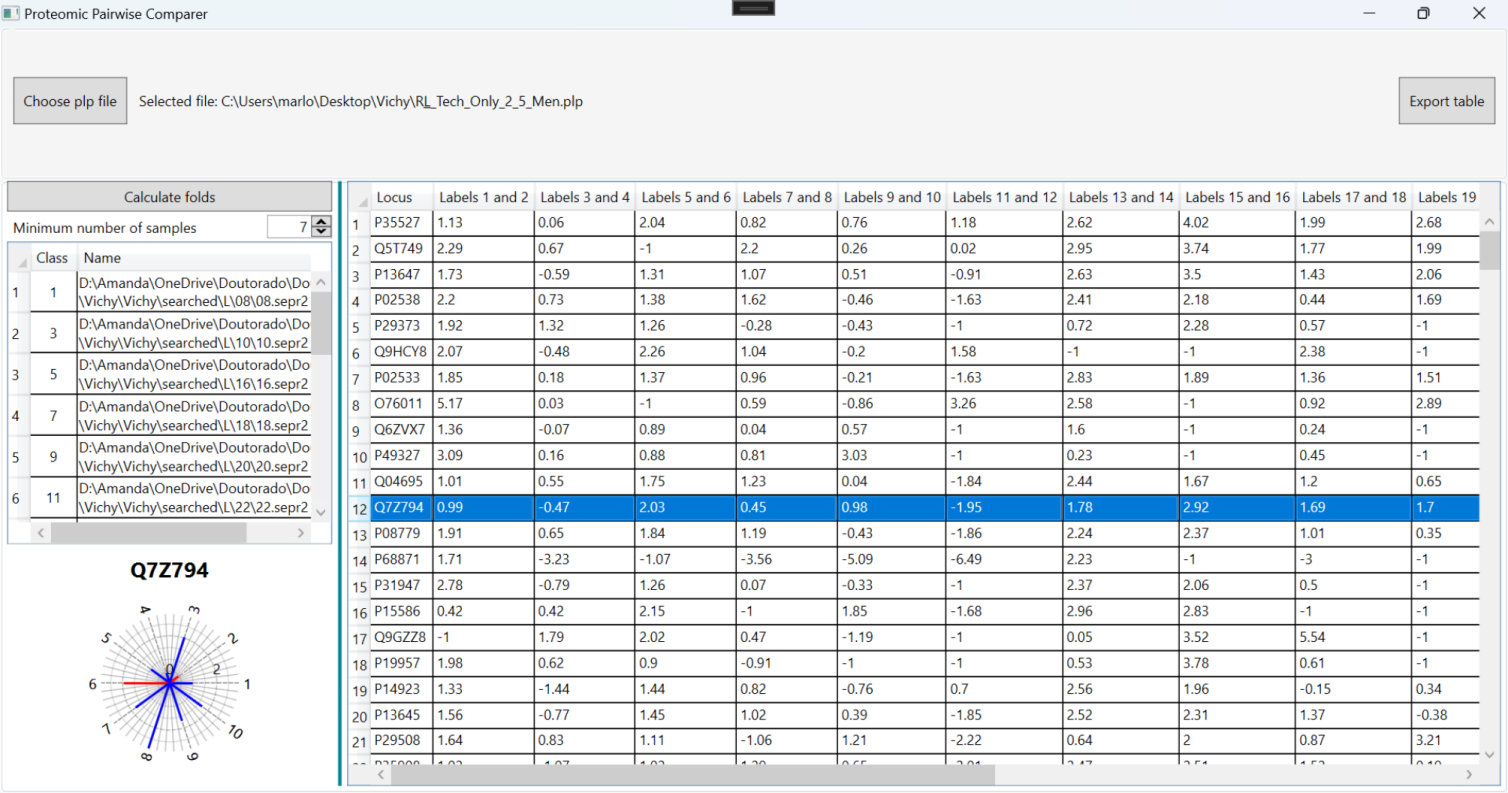
The Proteomic Pairwise Comparer software is designed for proteomic analysis, enabling the comparison of protein abundance in paired samples from the same individual under different conditions. By calculating fold changes and applying one-sample t-tests, it provides statistics on differential protein expressions, effectively addressing intra-individual variability. When selecting the protein in table the software shows the radar plot where blue lines are folding upregulated and red line down regulate for each paired sample.

The software’s central feature is its computation of fold changes—the ratio of protein abundance between paired conditions. It applies a one-sample t-test to these fold changes against a null hypothesis mean of zero, assuming no difference in protein expression between conditions. This process yields *p*-values alongside fold changes, providing a broad overview of the proteomic landscape. A visual representation of the software’s functionality is depicted in Fig. 1.

## Conclusion

We identified a set of proteins—Hemoglobin subunit beta (P68871), Keratin type I cytoskeletal 9 (P35527), Keratin type II cytoskeletal 1b (Q7Z794), F-box only protein 50 (Q6ZVX7), and Protein S100-A14 (Q9HCY8)—that were differentially abundant in both male and female cohorts with asymmetric sun exposure. These proteins indicate a defense mechanism against solar exposure, involving structural reinforcement, protein homeostasis, and cellular defense to counteract photoaging effects.

Our findings were enabled by the use of the Proteomic Pairwise Comparer software, which provides paired p-value analyses. This approach allowed for a more refined identification of proteins influenced by sun exposure, in contrast to non-paired analyses such as PatternLab’s T-Fold method, which did not consistently identify these proteins across datasets. This underscores the importance of paired experimental designs in proteomic studies, particularly when investigating human subjects, where inter-individual variability can obscure significant findings. When possible, opting for paired experimental designs allows for more sensitive statistical analyses to better explore and detect differentially abundant proteins.

## Electronic supplementary material

Below is the link to the electronic supplementary material.

Supplementary Material 1

Supplementary Material 2

Supplementary Material 3

## Data Availability

The mass spectrometry data is made available in the widely adopted proteomics repository, PRIDE. Details to enable the reviewer to assess our data while still not published are found below: Project accession: PXD050746 Reviewer account details: Username: reviewer_pxd050746@ebi.ac.ukPassword: 0sbg82yE.
