# Correction: Paired proteomic analysis reveals protein alterations in sun-exposed skin of professional drivers

**DOI:** 10.1038/s41598-025-04487-2

**Published:** 2025-06-20

**Authors:** Amanda C. Camillo-Andrade, Lucas A. Sales, Juliana S. G. Fischer, Rosario Duran, Marlon D. M. Santos, Paulo C. Carvalho

**Affiliations:** 1 Laboratory for Structural and Computational Proteomics, Carlos Chagas Institute, Rua Prof. Algacyr Munhoz Mader 3775, FiocruzCuritiba, Paraná Brazil; 2 Analytical Biochemistry and Proteomics Unit, Instituto de Investigaciones Biológicas Clemente Estable, Institut Pasteur de Montevideo, Montevideo, Uruguay; 3 Positivo University, Paraná, Brazil

Correction to: *Scientific Reports* 10.1038/s41598-024-82308-8, published online 31 March 2025

In the original version of this Article Amanda C. Camillo-Andrade and Lucas A. Sales were omitted as equally contributing authors. The Author Contributions section now reads:

“ACCA was responsible for participant selection, sample collection and preparation, data generation and analysis. MDMS contributed to sample preparation, data generation, and data analysis. LAS contributes to developing module and data analysis. JSFG participated in sample preparation. PCC and RD acted as advisors, overseeing the entire project, and contributing to discussions at all stages of the research. All authors participated in the writing of the manuscript. These authors contributed equally: Amanda C. Camillo-Andrade and Lucas A. Sales.”

The original Article has been corrected.

